# Supplementary figures and images for: Mechanistic insights into CAM-induced disruption of HBV capsids revealed by all-atom MD simulations
Source: PLoS Pathog. 2026 Feb 9;22(2):e1013566. doi: 10.1371/journal.ppat.1013566 (PMC12904591; doi:10.1371/journal.ppat.1013566)

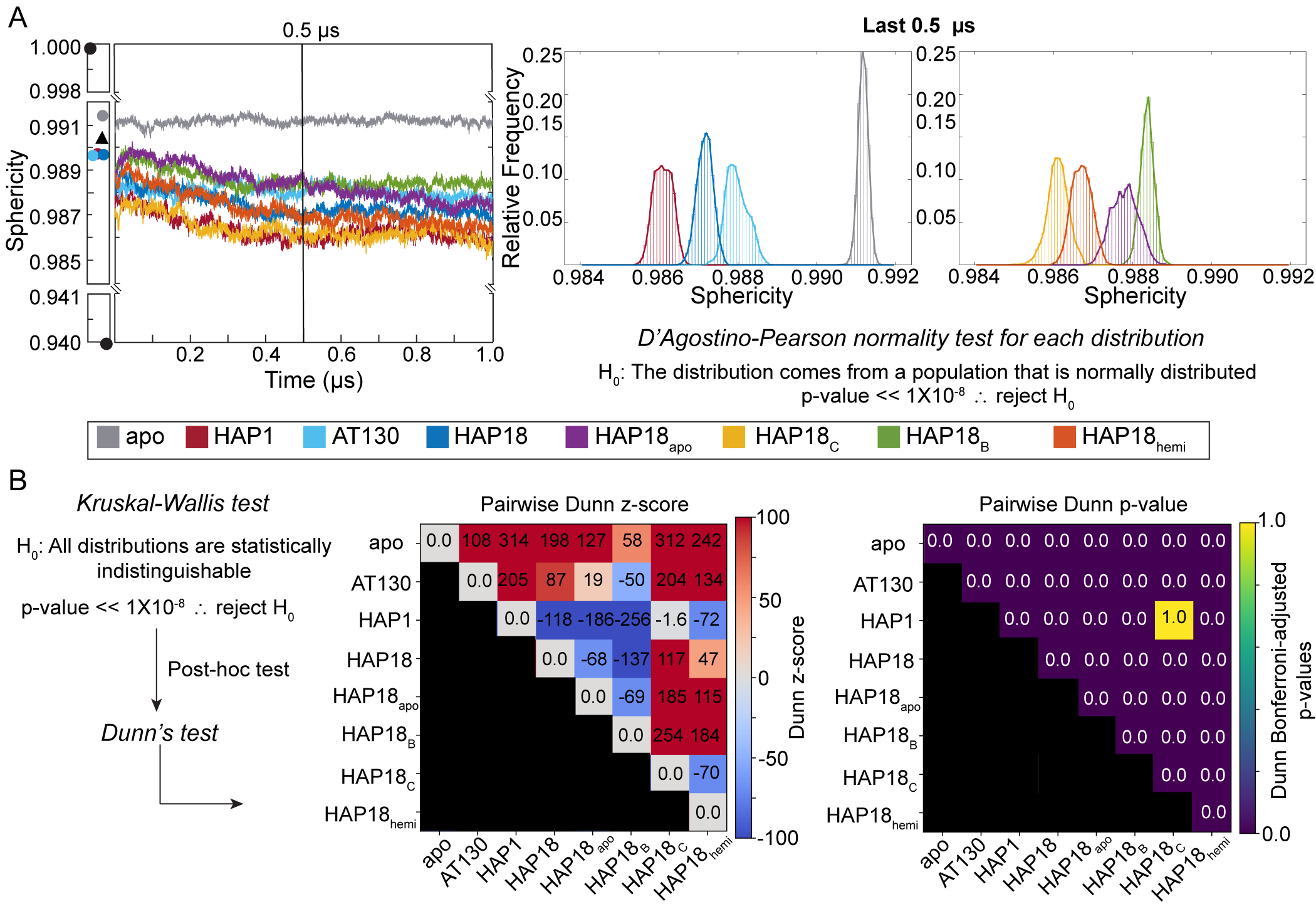

Supplement: S1 Fig — (A) Sphericity values were extracted from the last 500 ns of each simulation to minimize the influence of initial equilibration. Histograms of these values show minimal overlap between the apo-form and CAM-bound systems, with the apo-form displaying sphericity values not sampled among the other capsid simulations. To determine the appropriate statistical test for comparing sphericity across capsid systems, the normality of these distributions was assessed using the D’Agostino-Pearson test, which evaluates deviations in skewness and kurtosis from a normal distribution. The null hypothesis (H0) that each dataset follows a normal distribution was rejected for all systems (p-values «1X10−8). (B) Differences among sphericity distributions were evaluated using the Kruskal-Wallis test, a non-parametric test with the H0 that all sphericity distributions are statistically indistinguishable. Given an extremely small p-value (p-values «1X10−8), H0 was rejected meaning at least one sphericity distribution is statistically different. Pairwise comparisons were then performed using Dunn’s post-hoc test with Bonferroni correction, in which small absolute z-scores and adjusted p-values close to unity indicate that only the HAP1-bound and HAP18 C sphericity distributions are statistically indistinguishable. All other pairwise comparisons show statistically significant differences between distributions. (TIFF) [file ppat.1013566.s001.tif]

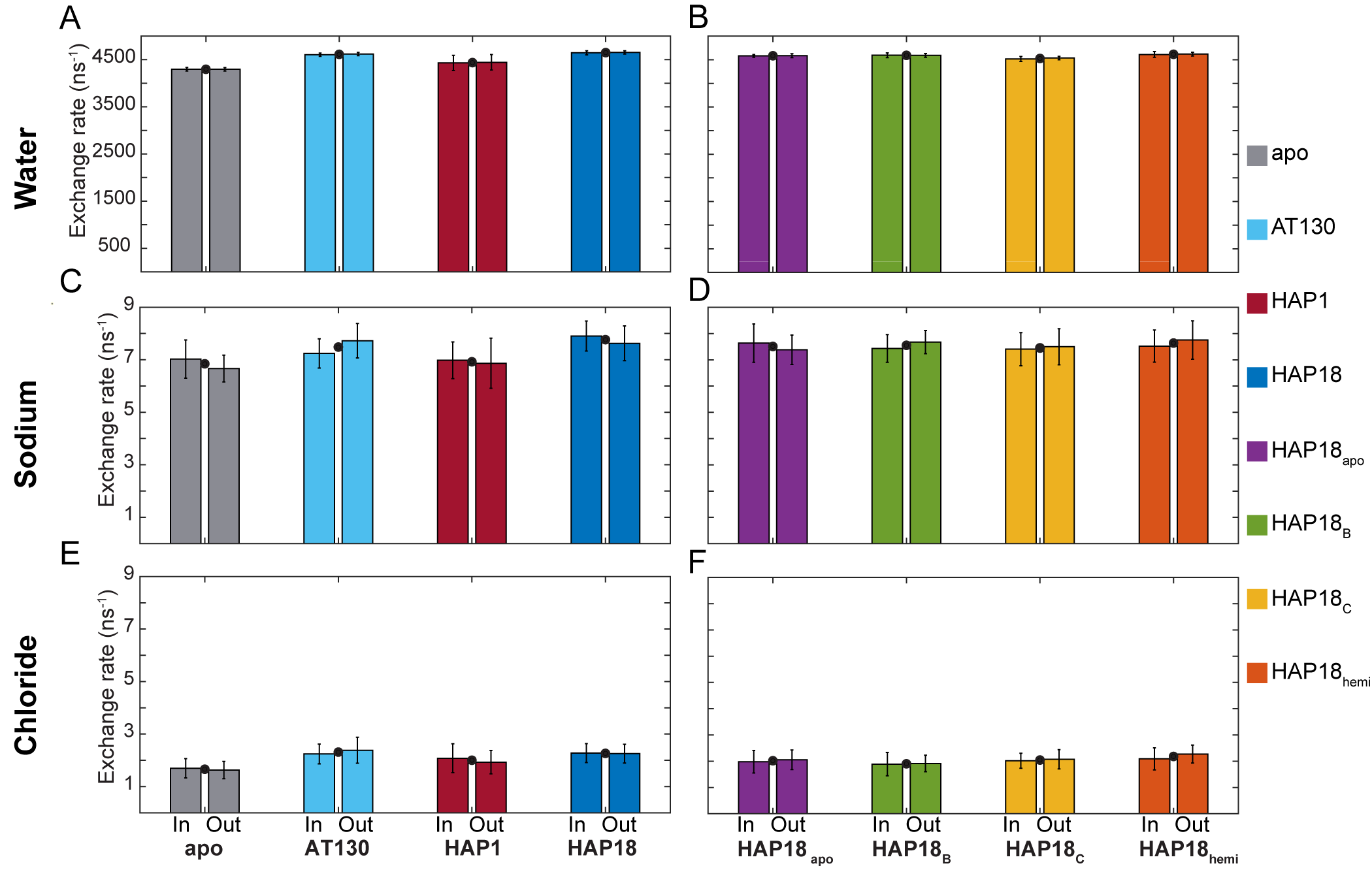

Supplement: S2 Fig — Inward (In) and outward (Out) exchange rates over the last 500 ns are shown for (A,C,E) apo-form, AT130-bound, HAP1-bound, and HAP18-bound systems, and (B,D,F) HAP18 apo, HAP18 B, HAP18 C, HAP18 hemi systems. Panels show (A-B) water, (C-D) sodium, and (E-F) chloride exchange rates. Black dots denote the mean between inward and outward rates per system; error bars represent standard deviations. (TIFF) [file ppat.1013566.s002.tif]

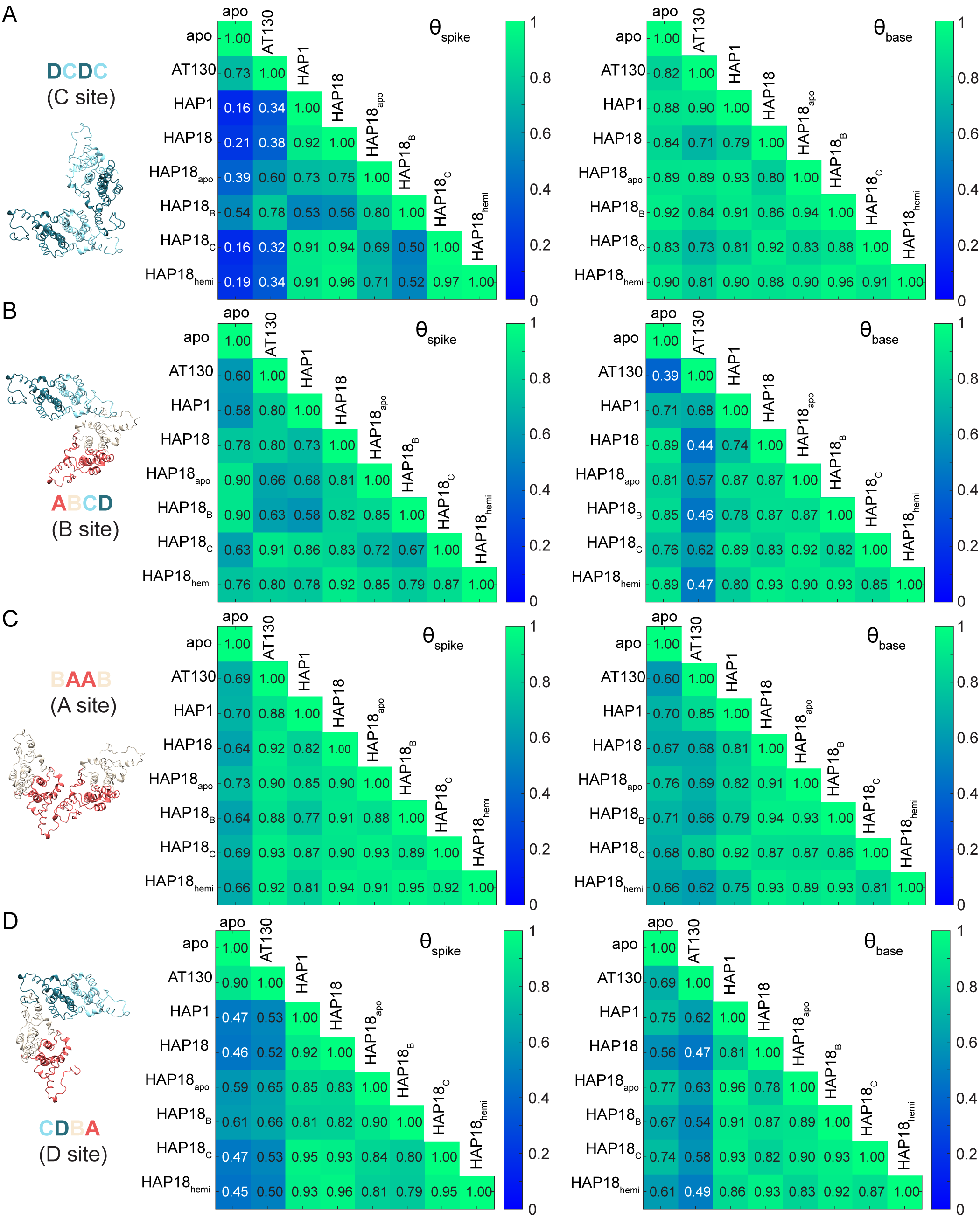

Supplement: S3 Fig — Scores are shown for each quasi-equivalent CAM-binding site: (A) C site, (B) B site, (C) A site, and (D) D site. Left panels show overlaps for θspike distributions, while right panels show overlaps for θbase distributions. Pairwise overlap scores are reported for all system combinations, where values near 0 indicate minimal similarity and values near 1 indicate high similarity between distributions. (TIFF) [file ppat.1013566.s003.tif]

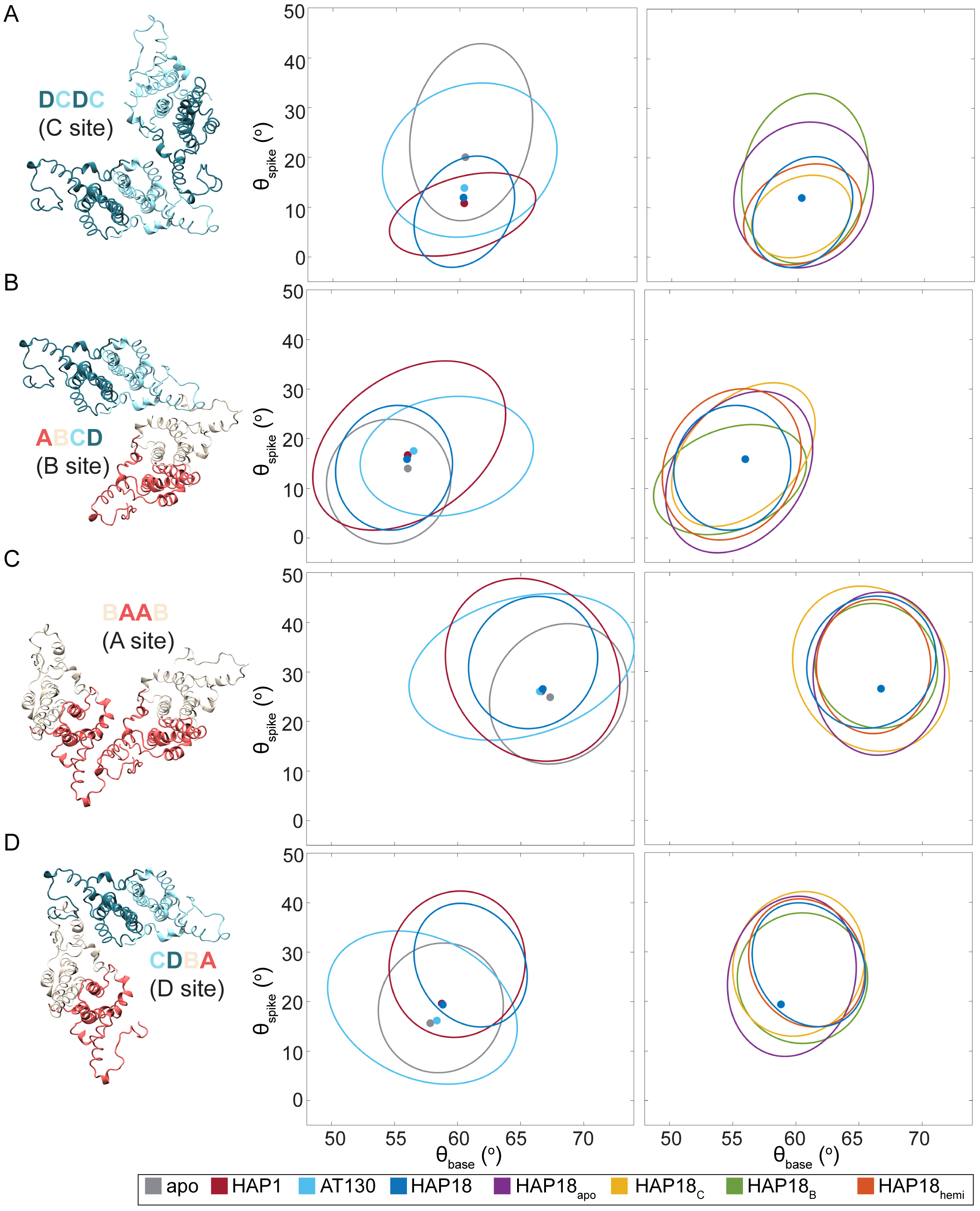

Supplement: S4 Fig — SDEs are shown for (A) C site, (B) B site, (C) A site, and (D) D site. Left panels correspond to apo-form, AT130-bound, HAP1-bound, and HAP18-bound systems, while the right panels show HAP18 apo, HAP18 B, HAP18 C, and HAP18 hemi systems. Each ellipse plots the relationship between θspike (y-axis) and θbase (x-axis), centered on the mean values. Ellipse radii reflect the standard deviation of each angle, and orientation is determined by the covariance between θspike and θbase. A 90% confidence level was used. (TIFF) [file ppat.1013566.s004.tif]

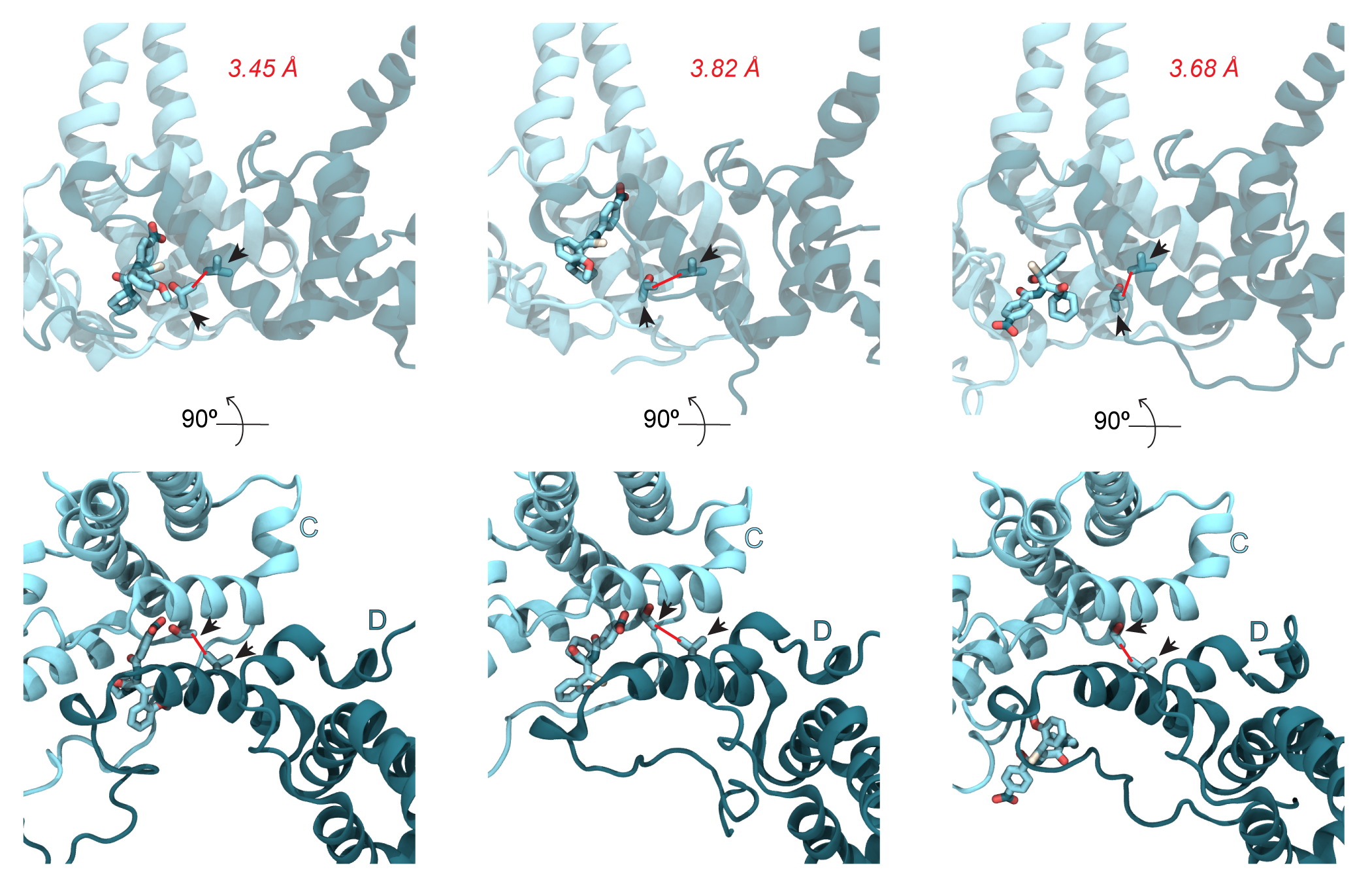

Supplement: S5 Fig — Extracted conformations from the AT130-bound capsid exhibit hydrophobic occlusion between T109 and V120, yet the AT130 molecule is still accommodated through distinct binding modes. Side chains of T109 and V120 are indicated with black arrows. The inter-residue distances are indicated above each conformation. (TIFF) [file ppat.1013566.s005.tif]

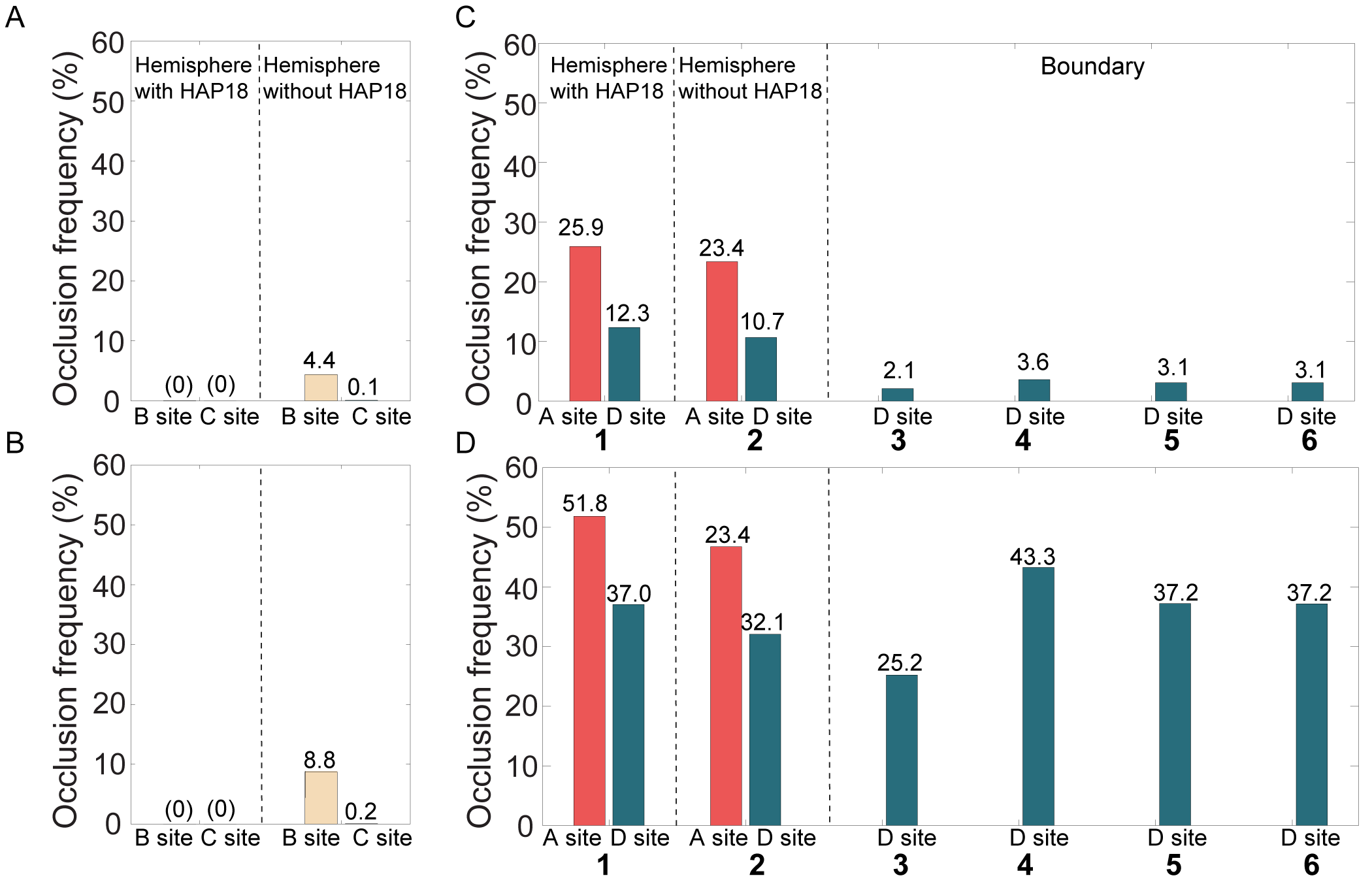

Supplement: S6 Fig — Contact frequencies across (A,B) B/C sites, and (C,D) A/D sites. Occlusion frequencies in (A,C) are normalized over 1.5 million conformations sampled over the last 500 ns of simulation, reflecting the cumulative sampling of 60 copies of each CAM site in a capsid conformation. Frequencies in (B,D) are recalculated by normalizing only over the conformations belonging to the same local environment, which improves direct comparison with full-capsid data. A, B, and C sites each display two local environments, corresponding to 30 of the 60 copies per capsid (750,000 conformations). D sites exhibit six local environments due to their boundary location. D sites at the hemispheres correspond to 20 of the 60 copies (500,000 conformations), while the four boundary environments correspond to 5 copies each (125,000 conformations per environment). (TIFF) [file ppat.1013566.s006.tif]

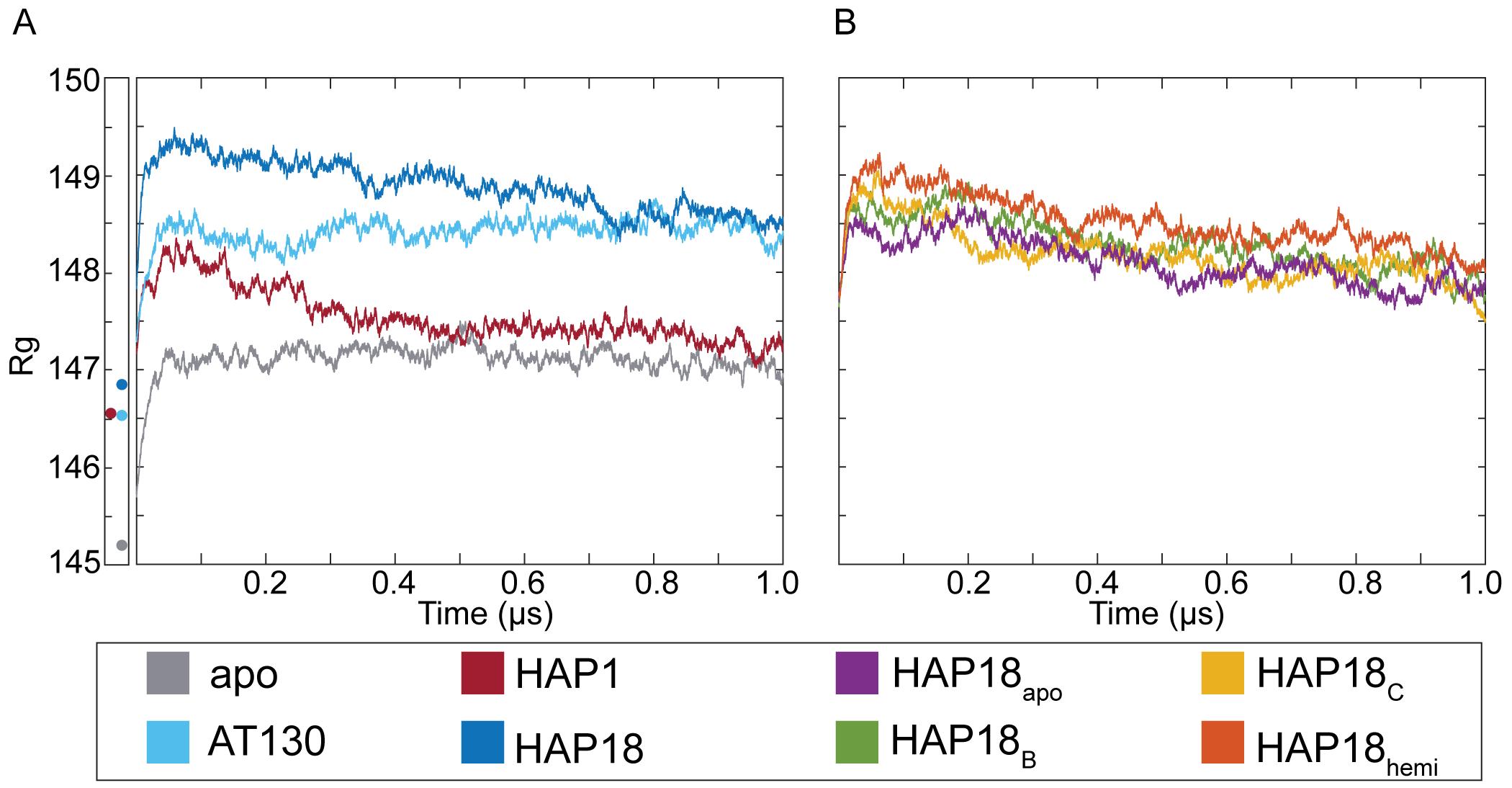

Supplement: S7 Fig — Rg over time for (A) apo-form, AT130-bound, HAP1-bound, HAP18-bound systems. Scatter points on the left indicate Rg values measured from the corresponding crystal structures.Time evolution of Rg for (B) HAP18 apo, HAP18 B, HAP18 C, and HAP18 hemi systems. (TIFF) [file ppat.1013566.s007.tif]

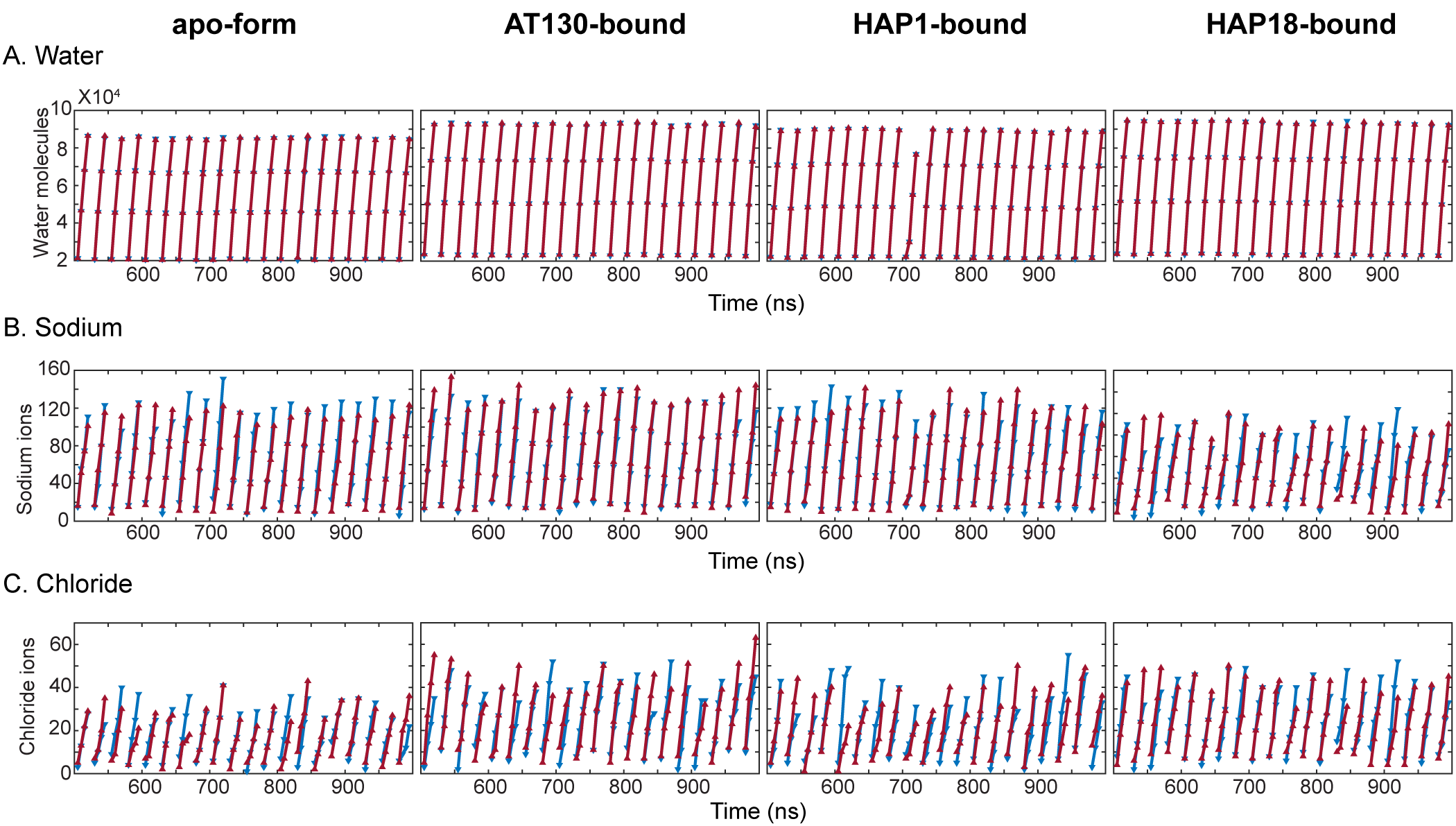

Supplement: S8 Fig — Number of (A) water molecules, (B) sodium ions, and (C) chloride ions. Exchange rates were calculated as the slopes of linear regressions fitted to data sampled every 5 ns within 25 ns intervals, with final rates taken as the average of all slopes. Inward and outward exchanges are shown in red and blue, respectively. Analyses were performed over the last 500 ns of each simulation. (TIFF) [file ppat.1013566.s008.tif]

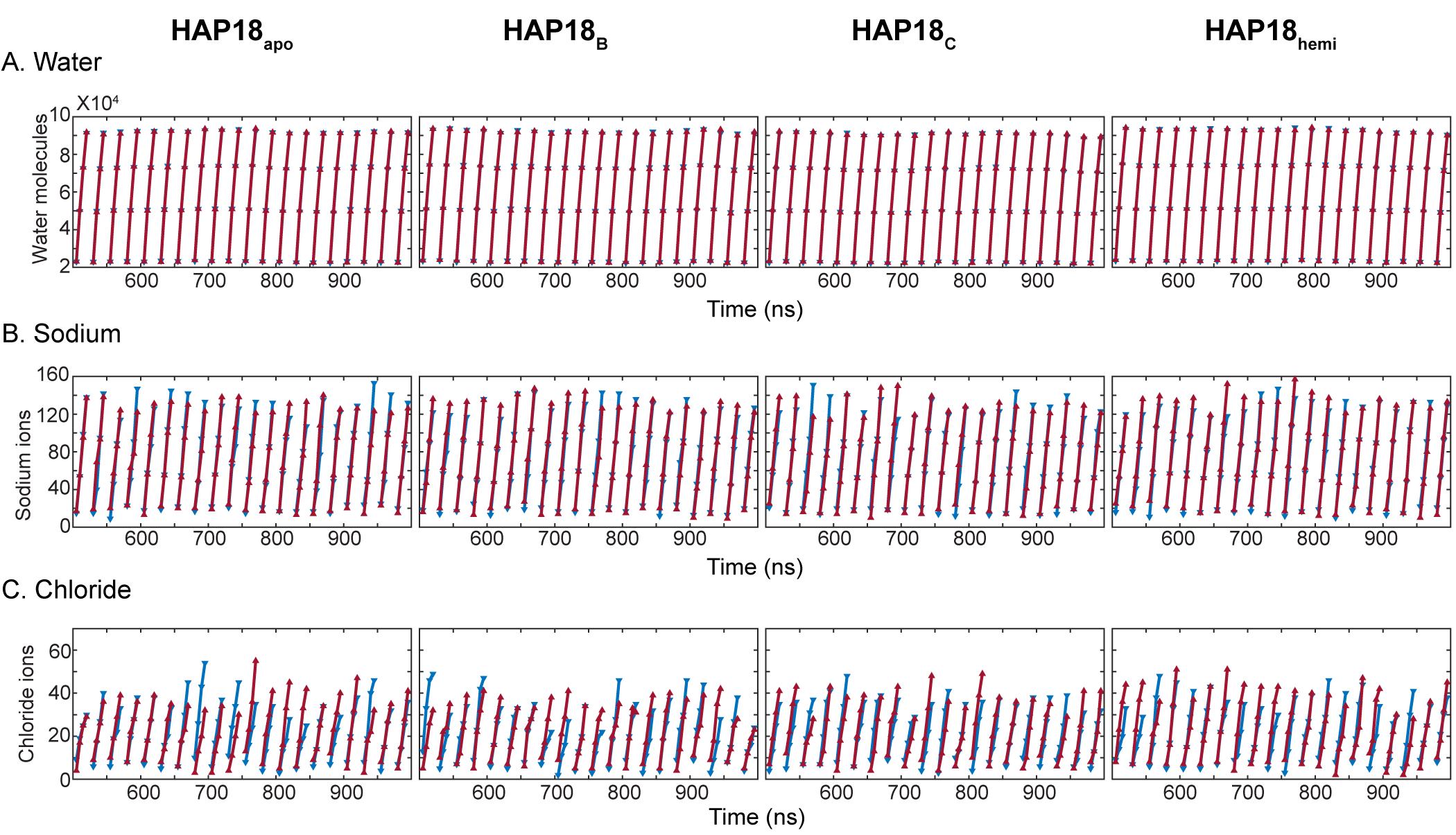

Supplement: S9 Fig — Number of (A) water molecules, (B) sodium ions, and (C) chloride ions. Exchange rates were calculated as the slopes of linear regressions fitted to data sampled every 5 ns within 25 ns intervals, with final rates taken as the average of all slopes. Inward and outward exchanges are shown in red and blue, respectively. Analyses were performed over the last 500 ns of each simulation. (TIFF) [file ppat.1013566.s009.tif]
