# Supplementary material for: Mechanistic insights into CAM-induced disruption of HBV capsids revealed by all-atom MD simulations
Source: PLoS Pathog. 2026 Feb 9;22(2):e1013566. doi: 10.1371/journal.ppat.1013566 (PMC12904591; doi:10.1371/journal.ppat.1013566)
Supplement: Attachment — Submitted filename: ReviewerResponse.pdf [file ppat.1013566.s010.pdf]

Response to Reviewers

Reviewers' Comments:

Reviewer's Responses to Questions

Part I - Summary

Please use this section to discuss strengths/weaknesses of study, novelty/significance, general execution and scholarship.

Reviewer #1: The authors employ extensive all-atom molecular dynamics simulations to explore how different capsid assembly modulators (CAMs) influence the structure and mechanical response of the hepatitis B virus capsid. The authors show that drug binding at specific interdimer interfaces transmits local strain through the lattice, producing distinct effects for each CAM class: CAM-A molecules (HAP1, HAP18) generate lattice distortion and destabilization, whereas the CAM-E molecule (AT130) preserves capsid geometry while locking it into a functionally inert state. The analysis also identifies B and C sites as central nodes of allosteric communication and offers a structural rationale for the cooperative drug-binding behavior reported experimentally.

Overall, the study provides a convincing atomistic explanation for how small molecules can either disrupt or over-stabilize the HBV capsid and contributes valuable mechanistic insight into antiviral strategies targeting core–protein interactions. I think that this is an interesting paper and I recommend it for publication, but only after the authors respond to my concerns raised below.

Reviewer #2: The paper from Perez-Segura et al. describes microsecond atomistic simulations of HBV capsids with various capsid assembly modulators (CAMs) bound. The authors find that different interfaces have distinct responses. The coupling between the interfaces redistributes the strain in such a way as to create "functionally allosteric" effects. These observations explain the known effects of different CAMs.

This is an impressive study. I have a few questions and suggestions for improvement.

1. On page 4 the authors discuss how long it takes for the capsid volume to stabilize. I would argue that it is difficult to determine that if only one simulation per system was performed. How confident are the authors that similar results would observed if the simulations were repeated?

The reviewer raises an important point – without replicates, we lack statistics about the timing of global volume equilibration across the different systems. Given a single run of each simulation, we have merely reported what we observed in terms of the approximate required volume relaxation timescales. We remind the reviewer that these timescales are simply a general characterization of how long it took for the capsid models to adapt to simulation

conditions when starting from the crystal coordinates. The magnitude of the change is much more informative (in that it suggests the degree to which the crystal is representative of the capsid under physiological conditions) than the timescale of relaxation from this perhaps arbitrary starting point. The apparent timescales needed for system equilibration would likely be different for alternative starting structures, simulation conditions, or equilibration protocol, yet are qualitatively informative here in terms of understanding the challenges all-atom MD simulations face when applied to large macromolecular complexes.

2. Figure 2 should be made clearer; see specific points a-d below.

a. I assume that all colors are the same as labeled in the F subplot? This should be explicitly stated in the caption.

We thank the reviewer for catching this oversight. Yes – the colors are defined in the legend in what was previously panel F (now panel J), and we have added the requested clarification to the figure caption.

b. For Figure 2A, I guess the $\times 10^7$ applies to the y-axis, but its positioning is less than ideal.

We thank the reviewer for the suggestion. We have included scientific notation in the y-axis labels to prevent any confusion.

c. For Figure 2D, the authors show a consistent decrease in sphericity for all CAM-bound capsids; however the difference is very small in comparison to the apo capsid, between 0.2-0.4%. How can we be sure that these differences are statistically significant?

We remind the reviewer that structural changes that appear insignificant on the scale of the complete capsid (36 nm in diameter) can be very meaningful on the scale of dimer-dimer interactions (<1 nm). Importantly, as noted in the manuscript, the CAM-A saturation levels used in our simulations are sufficient to induce capsid disassembly on experimental timescales, such that the Cp150 cross-link was required to maintain capsid integrity to obtain the CAM-bound crystals that served as our starting models for simulations. Thus, the structural changes we observed in response to bound CAMs are significant in that they appear to represent the early stages of shell disruption that leads to disassembly. It is also worth noting that sphericity ranges from 0-1 and remains above 0.9 for many geometric shapes one would not normally think of as sphere-like; for example an oblate ellipsoid with axes ratio 2:1:1 has sphericity around 0.9 while appearing elongated like a rugby ball. Sphericity is also independent of symmetry. This is why we augmented our analysis with a second metric, faceting, to more thoroughly characterize the morphological features of the conformational ensembles.

However, to address concerns regarding the statistical validity of our observations, we now include the results of statistical tests demonstrating that differences in sphericity values for all but two of the simulation ensembles are significant (see new Fig. S1). Interestingly, the two ensembles that are not statistically different are from the HAP1 and $HAP18_C$ bound cap-

sids, which are the only two systems containing CAM-As exclusively in C sites. Thus, these results further support our conclusion that the effect of CAM-As on C sites are meaningful when compared with the apo capsid, as well as reproducible across independent simulation models. We have added a new paragraph to the sphericity/faceting section to present these various points. We thank the reviewer for their suggestion, which provided the opportunity to strengthen the manuscript.

d. For Figure 2E, it would be very helpful to have an SI figure that illustrates the three angles measured here.

We thank the reviewer for highlighting the need for additional clarification regarding the definition of these angles. We now include a new panel in Fig. 2, which illustrates the geometric definition for the angles in the intact capsid.

3. On line 152, the subsection headline is "CAMs promote hexamer bending and asymmetry". While this seems to be true for CAM-A based on the presented data, the CAM-E results or AT130 are not discussed much in this subsection, and the data in Figure 3F shows the angle distributions to be very similar to the apo case. Therefore, would "CAM-As promote hexamer bending and asymmetry" be a better title and more representative of the described data?

We agree with the reviewer on this point and have made the suggested change to the subsection title.

4. Figure 7A and C, the authors should clearly label residues 124 and 120 here.

We have added the requested residue labels to the figure.

5. In discussion lines 670-672 the authors state: "Notably, occupancy of both B and C sites increases capsid volume regardless of CAM chemotype, whereas occupancy of B or C alone does not." I would argue that the paper only shows this for HAP18 and not generally for all CAMs. HAP1 was only simulated with C occupancy, and AT130 with both C and B, so we don't know if they would show the same trends as HAP18 for different occupancy choices. Also the HAP18 simulations were only performed once, so consistency of this result was not shown. The authors should add that this was only shown for HAP18 in the paper.

We value the reviewer's careful evaluation of our results. However, when drawing these conclusions, we consider what the MD results collectively indicate, in the context of available experimental data. The MD results do clearly show that occupancy of both B and C sites increases capsid volume for the case of AT130. Also, that the HAP1-bound capsid (B and C sites saturated in the experimental structure, but simulated with HAP only in C sites), initially trends toward the same volume state as AT130 yet then relaxes to the apo-form volume serves as compelling evidence that filled B/C sites also lead to an enhanced volume state for this CAM, while only filled C sites do not. This common enhanced volume state for capsids saturated either with CAM-Es or CAM-As is consistent with the trend

displayed by capsid crystal structures (Bourne et al. 2006 and Katen et al. 2013), providing validation for the MD observation, even in the absence of replicates.

Also, HAP18 is the largest and most structurally distortive CAM examined here. If HAP18 in either B or C sites does not lead to a volume increase (as shown by the MD results), then it is reasonable to conclude that smaller and less distortive CAMs like HAP1 and AT130 do not either. We now explicitly note this logic in the manuscript text:

> *Given that HAP18 is a large and highly distortive CAM, the finding that simultaneous B/C site occupancy is responsible for capsid size increase, rather than B or C sites alone, likely holds for smaller CAMs, as well as less structurally disruptive chemotypes.*

Further, given that HAP1 and $HAP18_C$ systems (filled C sites) display the same trend of relaxing toward the apo-capsid volume, we consider this result reproducible for HAPs across independent systems, similar to the outcome of simulation replicates. Taking our HAP1 versus HAP18 results together, we show that one HAP is not more or less morphologically distortive than the other on the timescale investigated. This observation makes sense because HAP1 and HAP18 share the same core and binding mode, with the latter containing an R-group that extends out of the mouth of the binding pocket. Given that multiple MD metrics throughout the paper indicate that B sites are CAM-ready, it makes sense that filling of B sites alone does not lead to capsid expansion. Given that multiple MD metrics throughout the paper also indicate that empty D sites can absorb C site distortion, it also makes sense that filling of C sites alone does not lead to capsid expansion. However, given the coupling of all three sites – B, C, and D – around the hexamer and their apparent cooperativity, it appears that filling of both B and C sites leads to volume expansion to accommodate the cumulative perturbations of the small molecules, regardless of chemotype, consistent with experimental structures.

6. In methods lines 784-785 the authors state that "Analogous sampling was extracted from previously published trajectories of the apo-form and AT130-bound capsids." Could the authors specify if there were any differences between these older simulations and the new ones with regards to force field and MD protocol?

We thank the reviewer for noting this subtle but important point. There are no differences between the older simulations and the new ones with regard to force field or MD protocol, and we have added a sentence in the Methods section to clearly state so.

Reviewer #3: This manuscript reports the results from all-atom molecular dynamics (MD) simulation studies of CAM binding of HBV capsids derived from Cp149. The study reveals the mechanism of CAM binding at the "HAP" pocket between Cp dimer-dimer interfaces to induce the structure changes and disassembly of intact capsids. Interestingly, the study revealed that each quasi-equivalent interface exhibits a unique response. While the A and D sites are unfilled, B sites are the most open and C sites are the hubs of allosteric control and the key CAM target, as their occupancy creates local distortion that is broadcast to adjacent sites, driving capsid faceting and destabilization. Interestingly, the extent of C

site adjustment and the nature of D site counterbalance vary with CAM chemotypes and underlines the distinct phenotypes of CAM-A and CAM-E on assembled capsids and highlights the allosteric (or tensegrity) nature of CAM action. The findings of this study shed light on the development of novel CAMs that can more efficiently induce the disassembly of nucleocapsids to inhibit HBV cccDNA synthesis.

Overall, the study is well conceived and executed. The results are clearly presented. The findings are of significance to understand CAM antiviral mechanisms and development of next generations of CAMs with improved pharmacological properties.

Part II – Major Issues: Key Experiments Required for Acceptance Please use this section to detail the key new experiments or modifications of existing experiments that should be absolutely required to validate study conclusions.

Generally, there should be no more than 3 such required experiments or major modifications for a "Major Revision" recommendation. If more than 3 experiments are necessary to validate the study conclusions, then you are encouraged to recommend "Reject".

Reviewer #1: N/A

Reviewer #2: (No Response)

Reviewer #3: Despite briefly discussed in the manuscript, it will be very interesting to determine the dynamic structure alteration and disassembly of capsids induced by dibenzothiazepines (DBTs) by all-atom molecular dynamics (MD) simulations.

We agree with the reviewer that using all-atom MD simulations to study the effects of DBTs on capsid structure and dynamics will be very interesting, and we plan to focus on DBT-bound capsids in an upcoming project.

Part III – Minor Issues: Editorial and Data Presentation Modifications Please use this section for editorial suggestions as well as relatively minor modifications of existing data that would enhance clarity.

Reviewer #1: 1. The abstract includes too much technical detail, especially about quasi-equivalent sites. For readers who are not familiar with this concept, the main idea is hard to follow. The abstract would be clearer if it focused on the overall findings and biological relevance instead of structural specifics.

We value the reviewer's feedback. However, the manuscript is specifically about our discovery that the quasi-equivalent CAM binding sites (A, B, C, and D) are coupled into a tensegrity network that CAMs can perturb, and the role of individual sites in this network. These are the mechanistic insights promised by the manuscript title. Without these terms, we cannot summarize the overall findings in the abstract in a way that makes the scientific advance apparent. Notably, this manuscript is not about biological relevance, but about how

small molecules alter the structure and dynamics of a macromolecular drug target. Thus, the findings that C sites are the most critical region to target for capsid disruption are essential to note in the abstract to attract the appropriate audience and maximize impact of the work. While the term quasi-equivalent is a key adjective to indicate how/why A, B, C, and D sites are different, all the reader needs to know to follow the abstract is that the four sites are different, which is communicated by note of each site's unique role in the tensegrity network. Finally, for readers who require a layman's summary, PLoS Pathogens includes a section that follows the abstract called the Author Summary, which is essentially a non-technical abstract. Our Author Summary is written for a broad, non-specialist audience, which should address the reviewer's concern while maintaining the original more descriptive abstract:

> *Hepatitis B is a major cause of chronic liver disease worldwide. The virus relies on a protein shell, called the capsid, to protect and deliver its genetic material to the host cell during infection. Some experimental drug molecules attack this shell, either forcing it to assemble incorrectly or breaking it apart after it has formed. To understand how these molecules work, we used powerful computer simulations to model the capsid at the level of individual atoms. We discovered that when molecules bind the capsid at certain sites, they create strain that spreads across the shell, sometimes leading to large distortions and instability. These insights explain how small molecules can disrupt the virus and point the way toward designing better antiviral therapies.*

2. The discussion of CAM-E molecules is confusing. They are described as accelerating assembly and producing morphologically normal but empty capsids, but the simulations use Cp149, which lacks the C-terminal domain and cannot encapsidate the genome. It is unclear whether the experimental data cited involve full-length core protein or genome-containing capsids. Please clarify this point.

We believe the reviewer is referring to the following sentence from the Introduction:

> *CAM-E molecules accelerate capsid formation, sabotaging pre-genome packaging to generate morphologically normal but empty particles.*

This is a statement indicating the mechanism of action of CAM-E molecules and explaining the origin of the "E for empty" nomenclature. The reviewer is correct that the C-terminal domain is required to bind the pre-genome and that Cp149 lacks this domain. Experiments with CAM-Es show that full-length Cp assembly is accelerated and the resulting capsids are empty and devoid of pgRNA. The Cp149 construct is commonly used to study CAMs *in vitro*, and experiments show that Cp149 assembly in the presence of CAM-Es is likewise accelerated, such that the model system is adequate to study the mechanism that would lead to packaging failure. To address the reviewer's concern, we now include references to experiments describing CAM-E effects on both Cp149 (without pre-genome) and Cp183 (with pre-genome): Delaney et al. 2002; Feld et al. 2007; Katen et al. 2010.

3. Can the authors compare and contrast their results with those of Tresset's group (ACS Nano 17, 12723–12733, 2023), which experimentally studied the energetics and kinetic assembly pathways of HBV capsids in the presence of CAMs?

We thank the reviewer for suggesting additional literature that examines the effects of CAMs on the HBV capsid. While the present manuscript does not present results related to assembly pathways that can be discussed in the context of the work by Tresset's group, we have now included mention of a finding by Tresset and co-workers that is consistent with our results and confers additional validity to our observations:

> *The finding that capsids saturated with CAM-Es exhibit enhanced volume, reduced sphericity, and minor morphological distortions relative to the apo-form is consistent with predictions from coarse-grain studies of HBV Cp assembly (Kra et al. 2023).*

4. Please clarify what is meant by the apo form. Does this refer to capsids without any bound CAM molecules, and were these systems equilibrated under the same simulation conditions as the CAM-bound ones?

The reviewer is correct, the apo-form refers to the capsid without any CAMs bound. The term "apo" by definition refers to a protein that is devoid of bound ligands or cofactors. Also, yes, all eight simulations were performed with identical conditions, and we now make a note of that in the Methods section.

5. It would be helpful to include representative structural snapshots for each case in Fig. 2 to visualize the morphological differences under various CAM-binding conditions.

We thank the reviewer for this suggestion to improve clarity of the manuscript. We have now augmented Fig. 2 with additional panels: Panel E highlights the definition of capsid faceting, one of the main morphological changes found in CAM-bound capsids. Panel F illustrates the conformational differences observed between the apo-form and CAM-bound capsids. Panel G shows how these morphological changes compare with idealized geometries, such as a perfect sphere or perfect icosahedron. We have further augmented the sphericity/faceting section to better explain both metrics.

6. The description of CAM-bound capsids as "more faceted" is not clear. Does this mean the particles show stronger icosahedral edges and faces, or that they deviate from icosahedral symmetry?

The reviewer's interpretation is correct. In this context, "more faceted" means that intact HBV capsids exhibit stronger icosahedral features, such as sharper icosahedral edges, protruded icosahedral vertices, and planar triangular icosahedral faces when CAM-As are bound. The description of CAM-A affected particles as "faceted" traces back to cryo-EM studies of HBV (*e.g.,* Schlicksup et al. 2018). We now include new panels in Fig. 2, which clearly illustrate the impact of CAM-As on so-called faceting angles and overall morphology as compared to a perfect sphere or perfect icosahedron.

7. The statement that "in the $T = 4$ capsid, hexamers are positioned on the icosahedral edges" is ambiguous. Does this mean the centers of hexamers lie along the edges connecting

adjacent pentamers?

The reviewer is correct about the meaning of icosahedral edges. We have clarified this meaning in the text using the definition the reviewer understood:

> *In the $T = 4$ capsid, hexamers are centered on the icosahedral edges, which connect adjacent pentamers on the icosahedral vertices.*

8. The angular parameters ϕ_5, ϕ_3, and ϕ_6 need to be defined more clearly, including the directions in which they are measured relative to the capsid center. A simple schematic showing how the faceting angle ($\phi_5 - \phi_3$) is determined would make this section easier to follow.

We thank the reviewer for highlighting the need for additional clarification regarding the definition of these angles. We now include a new panel in Fig. 2, which illustrates the geometric definition for the angles in the intact capsid.

9. The manuscript repeatedly refers to "allosteric control" and mentions that the dimer is "allosteric in the traditional sense." Please clarify what kind of allostery is meant—mechanical strain propagation across the lattice, conformational coupling within dimers, or both—and what evidence from the simulations supports this claim. Recent MD simulations (Science Advances 11, e ady7241, 2025) showed that allosteric coupling between capsid proteins promotes lattice elasticity but that complete T = 4 shells form only in the presence of the genome. It would help to comment on how genome-free Cp149 simulations relate to the allosteric and assembly behavior of genome-containing capsids.

In the following paragraph from Discussion, several sentences before stating that Cp "appears to be allosteric in the traditional sense," we provide the formal definition of allostery (what the reviewer calls conformational coupling), as well as the definition of tensegrity (what the revewer calls mechanical strain propagation across the lattice). Later in the paragraph, we state that *both* allostery and tensegrity are likely at work in the HBV capsid. The claim that Cp "appears to be allosteric in the traditional sense," is based on evidence from citations given at the end of the sentence, referencing both experimental work (Stray et al. 2004; Patterson et al. 2020) and our previous MD simulation studies (Zhao et al. 2020; Pérez-Segura et al. 2021). To clarify what is meant by "allosteric in the traditional sense," we now repeat identical language from the definition of allostery provided earlier in the paragraph (see bold text below). We hope the paragraph now addresses the reviewer's concerns:

> *Here, it is essential to distinguish between two complementary forms of inter-site communication. Formally, allostery refers to energetic coupling between binding sites, in which the complexation of a ligand in one site alters the conformational dynamics of a second, often distant site. However, not all long-range structural communication qualifies as allosteric in this sense. A related phenomena is tensegrity ("tensional integrity"), which involves mechanical coupling between sites: Local strain can be redistributed within a closed geometric shell, allowing systems like virus capsids to withstand acute structural deformation, while simultaneously communicating conformational changes from one location to another.*

> *The HBV capsid clearly exhibits tensegrity properties, including in response to CAM binding, and the propagation of strain induced by CAM occupancy represents a major mechanism underlying communication between quasi-equivalent interfaces. Given that CAM binding sites display cooperativity, the tensegrity effect functions practically as allostery with respect to small molecule uptake in the intact capsid.* ***The Cp dimer also appears to be allosteric in the traditional sense, exhibiting energetic coupling between the inter- and intradimer interfaces (Stray et al. 2004; Patterson et al., 2020; Zhao et al., 2020; Pérez-Segura et al., 2021).*** *In that case, tensegrity and allostery likely coexist in the HBV system, and may jointly mediate the structural effects of CAM binding on capsid assembly and stability. The observation that CAMs can exploit these mechanisms of conformational control over the capsid led to their early designation as core protein allosteric modulators (CpAMs), prior to revision of the nomenclature.*

With respect to commenting on how results for genome-free Cp149 capsids relate to the behavior of genome-containing capsids, we include the following text in the Discussion. This text presents the literature for experiments showing that CAM-induced disruption of empty Cp149 capsids is likewise relevant to Cp183 capsids that contain DNA, supporting the relevance of our findings to mature nucleocapsids:

> *The ability of CAMs to destabilize pre-formed capsids is of particular therapeutic relevance, as it provides a mechanism to impair genome-containing core particles that are critical to the HBV life cycle. While RNA-filled (immature) nucleocapsids exhibit enhanced mechanical stability due to counterbalancing interactions with the CTD and the enclosed pre-genome [Kim et al. 2022; Lecoq et al. 2023; He et al. 2024], and thus resist CAM-induced disruption, DNA-filled (mature) nucleocapsids are comparatively unstable and under internal stress that renders them more susceptible to morphological perturbation [Cui et al. 2013; Selzer et al. 2015; Dhason et al. 2012; Hu et al. 2017]. CAM-induced strain can therefore sensitize mature capsids to premature uncoating, blocking the delivery of the relaxed circular (rcDNA) to the nucleus and decreasing the formation of covalently closed circular DNA (cccDNA) [Guo et al., 2017; Taverniti et al., 2022].*

10. The manuscript states that "HAP molecules, as CAM-As, accelerate assembly but also misdirect it," but CAM-As are known to misdirect or destabilize assembly rather than accelerate it. In contrast, CAM-E molecules accelerate assembly and lead to empty but morphologically normal capsids. Since the simulations examine only pre-formed capsids, they cannot directly address assembly kinetics or misdirection. Please clarify that the results concern structural responses of mature capsids to CAM binding and separate the simulation findings from experimental conclusions.

We thank the reviewer for their careful reading of our manuscript, but it is accurate to say that CAM-As accelerate assembly. Based on reports in the literature (*e.g.,* Stray et al. 2005 and Bourne et al. 2008), CAM-As enhance the rate and extent of Cp assembly while

producing malformed or aberrant structures – CAM-As *both* accelerate and misdirect the assembly process. We agree that our simulations of pre-formed CAM-bound capsids cannot directly address assembly kinetics or misdirection, and nowhere in the manuscript do we make the claim that they can. The sentence the reviewer is referring to:

> *HAP molecules, as CAM-As, accelerate assembly but also misdirect it, producing aberrant products and destabilizing intact capsids.*

is a true statement used within the Discussion to remind readers of the mechanism of action of the CAM-A chemotype. We have added citations at the end of the sentence to clarify that it refers to the literature rather than findings from the present study. The very next sentence in the paragraph:

> *MD simulations indicate that HAPs "overfill" B and particularly C interfaces, such that accumulated strain propagates across the lattice and primes the shell for rupture.*

makes a summarizing statement about what the simulations show concerning the structural responses of complete capsids to bound CAMs, which we relate to the known ability of CAMs to induce shell destabilization, rupture, and ultimately disassembly. Rather than separating our findings from experimental conclusions, we contend that discussing MD results in the context of what is known from experiments is essential both to interpret our observations and to support the validity of our models.

11. The paper frequently refers to strain propagation and redistribution, but the terms "strain" and "stress" are not clearly defined. See, for example, ACS Nano 16, 317 (2022) for the experimental definition of stress in Dragnea's group's work. It would be helpful if the authors clarified whether these terms are used qualitatively or in a quantitative mechanical sense, and whether they correspond to measurable quantities such as local elastic strain or interfacial deformation. A brief clarification in the Methods or Discussion would make the tensegrity model easier to interpret.

We are grateful to the reviewer for checking our use of these terms and for suggesting relevant literature about stress distribution in coarse-grain models. Within the context of our manuscript, the term "strain" is used *qualitatively*, consistent with its use in prior literature describing CAM-induced deformation of the HBV capsid promoting destabilization and disassembly (*e.g.,* Schlicksup et al. 2020). We establish this qualitative definition in the Introduction section:

> *Structural studies have shown that CAM-A binding induces local strain on the shell, which leads to global destabilization that can trigger disassembly of metastable capsids.*

By contrast, the term "stress" appears only once in the manuscript, in the Discussion section, where the usage of the term is likewise *qualitative* and consistent with prior literature descriptions of the spring-loaded state of DNA-filled capsids (*e.g.,* Dhason et al. 2012):

DNA-filled (mature) nucleocapsids are comparatively unstable and under internal stress that renders them more susceptible to morphological perturbation.

However, to address the reviewer's concern, we have added a brief clarification in the Methods section as suggested:

The reversible response of the capsid to the presence or removal of bound CAMs was characterized in terms of local and global structural perturbations, as described below, rather than measuring elastic strain directly. Thus, the term "strain" is used in this work in a qualitative sense.

Reviewer #2: (No Response)

Reviewer #3: Line 36, because HBV is not a naked capsid virus, but an enveloped virus, the statement that "... leading to the production of non-infectious particles" is not accurate. It should be "... leading to the production of aberrant structures or empty capsids".

We thank the reviewer for their careful reading of our manuscript, however, the original statement is accurate. The reviewer is correct in noting that Cp assembly in the presence of CAMs leads to either aberrant structures or empty capsids. The next two sentences in the paragraph communicate these points, that CAM-As lead to aberrant structures, while CAM-Es lead to empty capsids:

By binding early-stage intermediates, CAMs alter the assembly process, leading to the production of non-infectious particles. These molecules are classified based on their assembly outcomes: CAM-A molecules accelerate and misdirect assembly to generate aberrant polymerization products, including hexamer sheets or tubes. CAM-E molecules accelerate capsid formation, sabotaging pre-genome packaging to generate morphologically normal but empty particles.

Based on their comment, it seems the reviewer's concern is related to the envelope of the extra-cellular HBV virion. We remind the reviewer that: (1) a portion of DNA-containing capsids newly assembled/matured in the cytoplasm re-infect the nucleus to maintain the nuclear cccDNA concentration, thus they are infectious despite being non-enveloped; (2) like DNA-containing capsids, empty capsids are commonly enveloped and exported by HBV as non-infectious particles, even in the absence of CAMs. See *e.g.,* Venkatakrishnan et al. 2016. Thus, a particle may be infectious in the cell although it lacks an envelope, and an enveloped particle may still be non-infectious. But a particle devoid of genome, either because it is structurally defective (induced by CAM-As) or simply empty (induced by CAM-Es), is always non-infectious. Importantly, "non-infectious" is a key word in the original statement, relevant to the consequences of CAM activity *in vivo*, beyond the assembly outcomes of Cp characterized *in vitro*. To address the reviewer's concern, while maintaining the authors' intended meaning, we have updated the text as follows:

By binding early-stage intermediates, CAMs alter the assembly process, leading to the production of particles that are non-infectious – either because they are structurally defective or devoid of genome.
